# Genome-wide identification and expression analysis of the response regulator gene family in alfalfa (*Medicago sativa* L.) reveals their multifarious roles in stress response

**DOI:** 10.3389/fpls.2023.1149880

**Published:** 2023-03-14

**Authors:** Yuqin Qiang, Xiaojuan He, Zhen Li, Siqi Li, Jia Zhang, Tao Liu, Mamateliy Tursunniyaz, Xinyu Wang, Zhipeng Liu, Longfa Fang

**Affiliations:** ^1^ State Key Laboratory of Herbage Improvement and Grassland Agro-ecosystems, College of Pastoral Agriculture Science and Technology, Lanzhou University, Lanzhou, China; ^2^ National Engineering Laboratory for Volatile Organic Compounds Pollution Control Material & Technology, University of Chinese Academy of Sciences, Beijing, China

**Keywords:** alfalfa, response regulators, stress response, gene family, expression analysis

## Abstract

As important components of the two-component regulatory system, response regulatory proteins (RRPs) play a crucial role in histidine phosphorylation-mediated signal transduction in response to environmental fluctuations. Accumulating evidence has revealed that RRPs play important roles in plant growth and stress response. However, the specific functions of *RR* genes (*RRs*) in cultivated alfalfa remain ambiguous. Therefore, in this study, we identified and characterized the *RR* family genes in the alfalfa genome using bioinformatics methods. Our analysis revealed 37 *RRs* in the alfalfa genome of Zhongmu No.1 that were unevenly distributed on the chromosomes. Cis-elements analysis revealed the involvement of *RRs* in responses to light, stress, and various plant hormones. Expression analysis of *RRs* in different tissues revealed their distinct tissue expression patterns. These findings provide preliminary insights into the roles of *RRs* in plant responses to abiotic stress, which can be used to improve the stress tolerance of autotetraploid-cultivated alfalfa plants *via* genetic engineering.

## Introduction

Adverse environmental conditions, such as drought, high salinity, and low temperatures (Hasanuzzaman et al., 2020; Imran et al., 2021), can greatly affect plant growth and crop production. To adapt to these challenges, plants have developed complex molecular response pathways for their survival (Razi and Muneer, 2021; Sun and Guo, 2022). The two-component system is one such response pathway that converts external stimuli into internal molecular signals in various prokaryotes, fungi, and plants (Hwang et al., 2002; Gao et al., 2008; Huo et al., 2020). The two-component system functions as a growth regulator during responses to stress factors, such as ethylene, cytokinins, light, and osmotic stress (Jain et al., 2008; Geng et al., 2022).

As important components of the two-component system, response regulators regulate the expression of downstream genes by receiving phosphate groups from receptors on conserved Asp residues in response to environmental stimuli (Kieber and Schaller, 2018; Hu et al., 2022). Various plant genome sequencing projects have improved our understanding of the functions of response regulatory (*RR*) genes in plants (Zhang et al., 2012). RRPs are encoded by a large gene family in plants (Wang et al., 2007), which can be divided into three subtypes based on their domain structure and amino acid sequence: A, B, and P. Type-A *RRs* only possess a receiver domain, and their transcripts are induced by cytokinins and nitrates (West and Stock, 2001; Hass et al., 2004). Type-B *RRs* are transcription factors that can activate their target genes, including type-A *RRs*, and act as positive regulators of the cytokinins response (Mason et al., 2005). Type-P *RRs* lack the Asp phosphorylation sites required to maintain their activity and may affect the biological clock (McClung, 2006).

To date, *RRs* have been identified on a genome-wide scale in various plant species, such as *Arabidopsis thaliana* (D’Agostino et al., 2000), *Oryza sativa* (Tsai et al., 2012), *Zea mays* (Wang et al., 2022), *Gossypium hirsutum* (Zhao et al., 2022), and *Glycine max* (Le et al., 2011). Although the number of identified *RRs* varies among plants, their functions are conserved in these different species. Many stress tolerance-related genes have been identified in different plants. For instance, *OsRR6, GmRR01*, *GmRR02*, *GmRR25*, and *GmRR32* have been reported as positive regulators of stress response, increasing the plant resistance to drought and salt stress (Le et al., 2011). In contrast, *OsRR9* and *OsRR10* negatively regulate salt tolerance (Wang et al., 2019; Bhaskar et al., 2021). *Z. mays ZmRR1* is the only gene reported to be involved in cold tolerance (Zeng et al., 2021). Some *RRs* have been found to play vital roles in developmental and environmental signals mediated by light, cytokinins, and ethylene (Doi et al., 2004; Ito and Kurata, 2006). For instance, the expression patterns of *O. sativa Ehd1* and *ZmRR3* are related to cytokinins (Pareek et al., 2006), and those of *GhRR41*, *SlPRR1*, *SlPRR2*, *SlPRR3*, *SlPRR4*, *SlPRR5*, and *SlPRR6* are mainly related to the circadian rhythm and regulation of flowering time in plants (He et al., 2016).

Alfalfa, the most widely cultivated legume forage in the world with a planting area of 32 million hectares (Ren et al., 2021), is known as the “queen of forage” because of its high nutritional value as a source of proteins, vitamins, and other nutrients for livestock. It also exhibits potential for ethanol production (Liu et al., 2021). Alfalfa is widely cultivated in the arid and semiarid areas of the Northeast, Northwest, and Central North regions of China, and its production is strongly constrained by various biotic and abiotic stresses (Dong et al., 2023). Although the stress-tolerance-related functions of *RRs* have been widely reported in other crops, only a few studies have investigated their roles in the stress response of cultivated alfalfa plants. Therefore, in this study, we systematically identified and characterized the *RRs* to understand their biological functions, physicochemical properties, gene structures, evolutionary relationships, and location on the chromosome. We also analyzed the expression patterns of *RRs* under different abiotic stresses using quantitative reverse transcription-polymerase chain reaction (qRT-PCR). Our results provide a basic understanding of the roles of *RRs* in cultivated alfalfa and lay the foundation for further breeding studies of this plant *via* genetic engineering.

## Materials and methods

### Plant growth, treatments, and tissue collection

Alfalfa (Zhongmu No.1) seeds from the College of Grassland Agricultural Science and Technology of Lanzhou University (humidity: 80%) were germinated in a culture dish and grown in half-concentration Murashige and Skoog nutrient solution (pH 5.8) under 16/8 h light/dark conditions at 22 °C and 80% relative humidity for 15 d. For the stress treatment, fifteen groups of alfalfa seedlings were separately treated with abscisic acid (ABA; 10 µM), NaCl (250 mM), and mannitol (400 mM) for different time points (0, 1, 3, 6, and 24 h), and each treatment group contained five plants. Whole plants from different treatments were separately stored at –80 °C in an ultra-low temperature refrigerator for subsequent analysis. Total RNA was extracted with the TRIzol Total RNA Extraction Kit (Shenggong Bioengineering Co., Ltd., Shanghai), and reverse transcribed into cDNA using the TIANScript II RT Kit (Tiangen Biochemical Technology Co., Ltd., Beijing) for gene expression verification.

### Identification of *RR*s in alfalfa

Alfalfa genome coding sequence (CDS) and protein sequences were downloaded from the website (http://47.92.172.28:12088/). RR protein sequences of *A. thaliana* from the TAIR (https://www.arabidopsis.org/) were used as query sequences to identify candidate *RRs* in the alfalfa genome using the Protein Basic Local Align Search Tool (BLASTP) with an E-value cutoff of 10^-5^. A hidden Markov model (HMM) for the response regulator receiver domain, PF00072, from the PFAM protein database (http://pfam.xfam.org/) was used to further filter the previously identified *RRs*, and the remaining protein sequences were considered to be members of *RR* gene family.

### Gene characteristics, structure, conserved motif, and subcellular localization analyses

Physical parameters of putative proteins, including the lengths of amino acid sequences, molecular weights (MWs), theoretical isoelectric points (pIs), and instability index, were calculated using the online ExPASy tool (http://www.expasy.org/tools/protparam.html), and the gene structure display server program (http://gsds.cbi.pku.edu.cn/) was used to illustrate the exon–intron structures by alignment of the CDSs of individual *RRs*. For motif structures analysis of RRPs, the online MEME program (http://meme-suite.org/tools/meme) was used with the default parameters, and the optimum motif widths were set at 6 residues. For subcellular localization analyses, the CELLO webtool (http://cello.life.nctu.edu.tw/) was used to predict the subcellular location of RRPs.

### Chromosomal localization and collinearity analysis of *RRs*

To better recognize the genomic distribution of *RRs*, the R package RIdiogram was used to draft the chromosomal location map of *RRs* based on the genome annotation files of alfalfa. For gene duplication event analysis, we used the DupGen_finder pipeline (https://github.com/qiao-xin/DupGen_finder) to identify different modes of gene duplication (tandem duplication [TD], transposed duplication [TRD], whole genome duplication [WGD], proximal duplication [PD], and dispersed duplication [DSD]) using the default settings (Qiao et al., 2019). For Ka, Ks, and Ka/Ks calculations, the KaKs_Calculator 2.0 was used based on the Tamura–Nei model model (Wang et al., 2010).

### Phylogenetic and cis-regulatory element analyses

To analyze the evolutionary relationships among *RRs* in alfalfa, a phylogenetic tree containing 37 alfalfa *RRs* and 32 Arabidopsis *RRs* was constructed with MEGA software (version 6.0; https://www.megasoftware.net/egasoftware.net) using the neighbor-joining method, and the bootstrap coefficient was set to 1000 times. PlantCARE database (http://bioinformatics.psb.ugent.be/webtools/plantcare/html/) was used to determine potential cis-regulatory elements within 2000 bp promoter sequences of *RRs*.

### Expression analysis of *RRs*

Transcriptome data from the CADL-Gene Expression Atlas (https://www.alfalfatoolbox.org/) and National Center for Biotechnology Information Sequence Read Archive database (http://www.ncbi.nlm.nih.gov; accession numbers SRX4079528–SRX4079572) were used to explore the expression profiles of *RRs* in different tissues (leaves, flowers, pre-elongated stems, elongated stems, roots, and nodules) and with different treatments (ABA, NaCl, and mannitol). Subsequently, the expression patterns of three *RRs* under different treatments in alfalfa were explored using the 2 x SG Fast qPCR Master Mix (Sangon Biotech, Shanghai, China) on a CFX96 Real-Time PCR Detection System (Bio-Rad, Los Angeles, CA, USA). The reaction system consisted of 10 μL volume (0.2 μL of each primer, 2.6 μL of ddH_2_O, and 1 μL of DAF buffer, 1 μL of cDNA, and 5 μL of 2 x SG Fast qPCR Master Mix). PCR reaction conditions were as follows: 94 °C for 30 s, followed by 20 cycles of 94 °C for 5 s and 54 °C for 30 s. The actin gene from alfalfa was selected as an internal reference, and the relative expression level of each gene was calculated using the 2^-ΔΔCT^ method. Three biological replicates were used, and the significant differences between the treatment and control groups were determined at the same time point using a *t*-test. A histogram with error lines was drawn using R package ggplot2. All qRT-PCR validation primers used in the present study are listed in **Table S1**.

## Results

### Identification and gene characteristic analyses of the *RR* gene family in alfalfa

We identified 37 *RRs* in alfalfa based on a homology-based prediction approach using BLASTP. Further analysis of the evolutionary relationship between the structural specificity and conservation of these *RRs* led to the identification of 10 type-A, 16 type-B, and 11 type-P *RRs* (**Table 1**). The number of *RRs* was close to the *RR* gene family size observed in other plant species, such as *A. thaliana* (32 genes) and *O. sativa* (36 genes) (Ramírez-Carvajal et al., 2008).

**Table 1:** Details of the *RRs* identified in alfalfa.

Gene name	Protein length	Protein MW(Da)	PI	Protein GRAVY	instability index
MsG0780040812	139	15954.74	8.06	-0.116	37.32
MsG0180002532	183	20517.76	5.39	-0.256	55.27
MsG0180002564	369	42036.46	6.31	-0.512	29.97
MsG0780040837	130	14494.9	5.67	-0.127	40
MsG0580025893	226	24373.56	4.56	-0.137	52.87
MsG0380016661	490	54145.17	6.78	-0.502	54.59
MsG0180000565	298	34120.53	6.08	-0.702	37.39
MsG0780040418	238	26912.11	5.56	-0.763	55.33
MsG0880047619	602	67252.45	5.17	-0.555	55.22
MsG0180000563	523	59185.05	6.78	-0.615	43.42
MsG0880047620	1214	134882.9	5.04	-0.488	49.88
MsG0380016366	262	29336.13	4.54	-0.356	65.92
MsG0380012160	167	18959.93	5.96	-0.292	47.49
MsG0480022917	549	62653.28	5.8	-0.438	41.81
MsG0880047126	680	74037.8	5.75	-0.532	42.23
MsG0880046376	212	23428.01	5.84	-0.194	55.14
MsG0080047933	765	86289.36	6.24	-0.676	50.98
MsG0780041771	510	57843.29	7.16	-0.663	47.57
MsG0280008856	666	73132.27	5.93	-0.599	48.59
MsG0380017187	550	63107.1	6.05	-0.421	44.84
MsG0580027346	313	36412.24	5.78	-0.662	39.58
MsG0380013071	854	97011.48	7.17	-0.687	55.34
MsG0180002180	196	21756.03	5.53	-0.289	63.63
MsG0880042893	666	75235.66	6.14	-0.797	53.22
MsG0480019003	225	25694.76	6.5	-0.199	41.65
MsG0480020869	760	83243.94	6.09	-0.683	40.35
MsG0880045009	144	16167.73	6.3	-0.377	32.33
MsG0280007899	637	70612.32	7.3	-0.549	49.58
MsG0880043607	177	20076.19	5.55	-0.369	46.91
MsG0780041732	291	33207.86	8.27	-0.719	39
MsG0380014518	191	21446.43	5.97	-0.373	45.41
MsG0380016233	521	59588.48	5.76	-0.573	50.3
MsG0380015850	201	21795.19	6.61	-0.068	56.41
MsG0380016710	155	17090.81	6.59	-0.312	44.74
MsG0880045487	372	41397.7	6.95	-0.391	36.7
MsG0780040815	187	20432.41	5.8	-0.426	35.86
MsG0380017376	642	70348.95	6.15	-0.487	45.85

In addition to the identification of *RRs* in alfalfa, we characterized the physicochemical properties of RRPs. Detailed information is provided in **Table 1**. The lengths of the identified RRPs ranged from 130 to 1214 amino acids (AAs), and the pIs varied from 4.54 to 8.27 (**Table 1**). These RRPs also displayed a wide range of MWs from 14.49 to 134.88 kDa (**Table 1**). Notably, most of these proteins were soluble hydrophilic proteins as the overall mean hydropathicity for all RRPs was negative (< 0) (**Table 1**).

### Phylogenetic, gene structure, and protein domain analyses of RRPs

**Figure 1:**
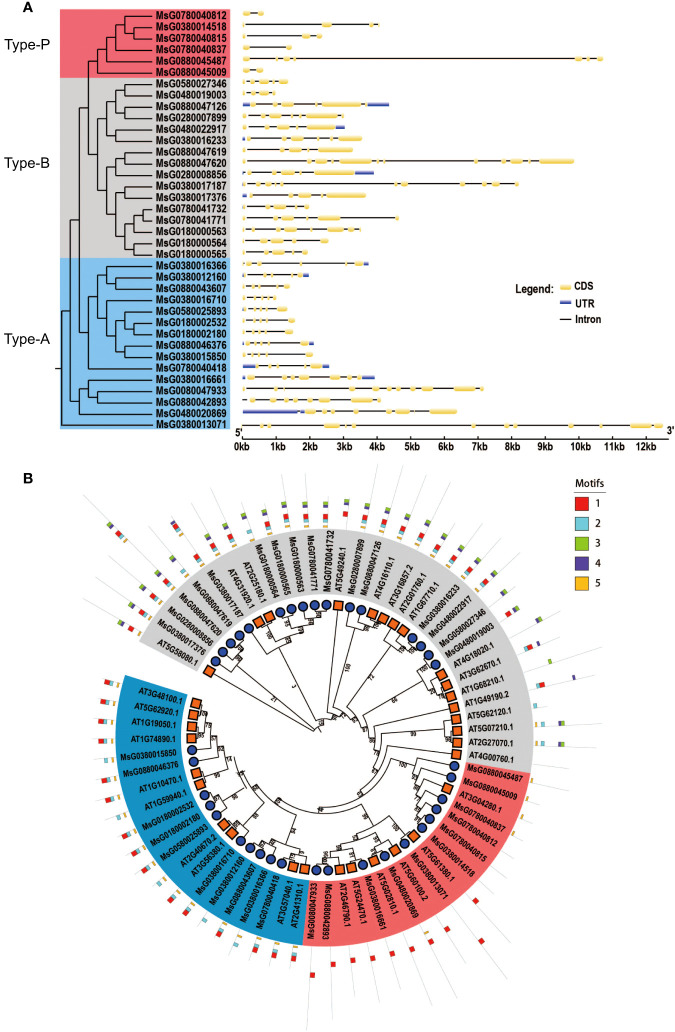
Gene structure, phylogenetic tree and meme structure analyses of *RRs*, Blue, type-A; Gray, type-B; Pink, type-P. **(A)** The rooted phylogenetic tree was constructed with 37 RR amino acid sequences of alfalfa. Exon-intron organization of corresponding of the *RRs*. The exons and introns are represented by yellow boxes and black lines, untranslated regions (UTRs) are represented by blue boxes, respectively. The length of each *RR* gene is shown as a proportion. **(B)** Phylogenetic tree and meme structure of *RRs* between alfalfa and *A. thaliana*. The phylogenetic tree was constructed using MEGA 6.0 by the neighbor-joining method with 1000 bootstrap replicates. The tree was divided into 3 subgroups. Members of different subgroups are denoted by solid circles with red, blue and gray colors, respectively. The conserved motifs in the RPPs were identified by MEME. The black lines represent no conserved sequences. Each motif is indicated by a colored box numbered at the bottom. Details are listed in **Figure S1**.

To confirm the evolutionary relationships among RRPs, we aligned the full-length RRPs of alfalfa and *A. thaliana* to construct a phylogenetic tree using MEGA 6.0. Based on the phylogenetic tree, 69 *RRs* (32 *RRs* in *A. thaliana* and 37 *RRs* in alfalfa) were divided into three subfamilies: 20 type-A, 32 type-B, and 17 type P-*RRs* (**Figure 1B**). The structural diversity of genes drives the evolution of multigene families. To better understand the structural diversity of *RRs*, we plotted exon–intron organization maps and found that all *RRs* contained more than one intron. The number of exons in the 37 genes varied from 2 to 12. Some genes, such as *MsG0880047620*, *MsG0380017187*, and *MsG0380013071*, contained more than 10 exons, and others had less than 10 introns. Notably, some type-A and type-B *RRs* contained untranslated regions (UTRs), but none of the type-P *RRs* had any UTRs (**Figure 1A**).

To further investigate the distribution and structural diversification of conserved motifs in alfalfa RRPs, we used the MEME software and found that most closely related members shared common motifs, but the conserved motifs among the subfamilies were significantly different, with some exceptions. For example, all type-A RRPs, except MsG0380016366, MsG0380012160, and MsG0380015850, contained motifs 1, 2, and 5; all type-B RRPs, except MsG0880047619, MsG0180000564, MsG0580027346, and MsG0480019003, contained motifs 1, 2, 3, 4, and 5 (**Figure 1B**; **Figure S1**).

### Chromosomal distribution and subcellular localization of *RRs*

**Figure 2:**
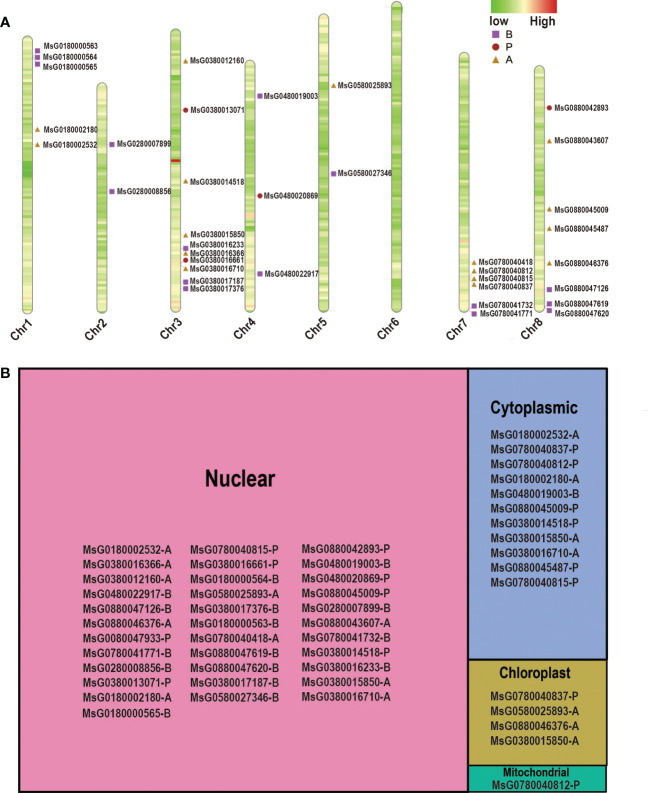
Chromosome distribution and subcellular localization of RRPs. **(A)** Chromosome distributions of different subtypes RRPs in alfalfa. The chromosome number is indicated at the bottom of each bar. **(B)** Tree map showing the subcellular localization of different subtypes RRPs in alfalfa.

To study the orientation of *RRs* on each chromosome, we obtained accurate information about the initiation sites to construct the chromosomal location map (**Figure 2A**). Our results showed that the *RR* family members were distributed unevenly across the eight chromosomes of alfalfa, with the number of *RRs* varying among the chromosomes. Almost half of the *RRs* (18 *RRs*) were present on Chr3 and Chr8 of alfalfa, but there were no *RR* genes on Chr6, and *RRs* were less distributed on Chr2, Chr4, and Chr5, with only two, three, and two genes found in these chromosomes, respectively. Interestingly, most of the *RRs* were located in the distal centromere region, indicating that these *RRs* may have experienced long-term evolution in alfalfa.

Information on subcellular localization is crucial for understanding the functions of proteins. The CELLO web server was used to determine the subcellular localization of RRPs. We found that most RRPs were mainly located in the nucleus and mitochondria (**Figure 2B**). This is not surprising as some RRPs can function as transcription factors. Furthermore, only a few *RR* members were predicted to be located in the chloroplast and one was found to be targeted to the mitochondria. Notably, some RRPs exhibit more than one subcellular localization. For example, MsG0780040812 is located in both the mitochondria and cytoplasm, whereas MsG0380015850 is distributed in almost all subcellular compartments, except for the mitochondria (**Figure 2B**). Therefore, these proteins may need to be trafficked to different subcellular organelles under different conditions to perform their functions.

### Cis-acting element analysis of the *RR* promoter

**Figure 3:**
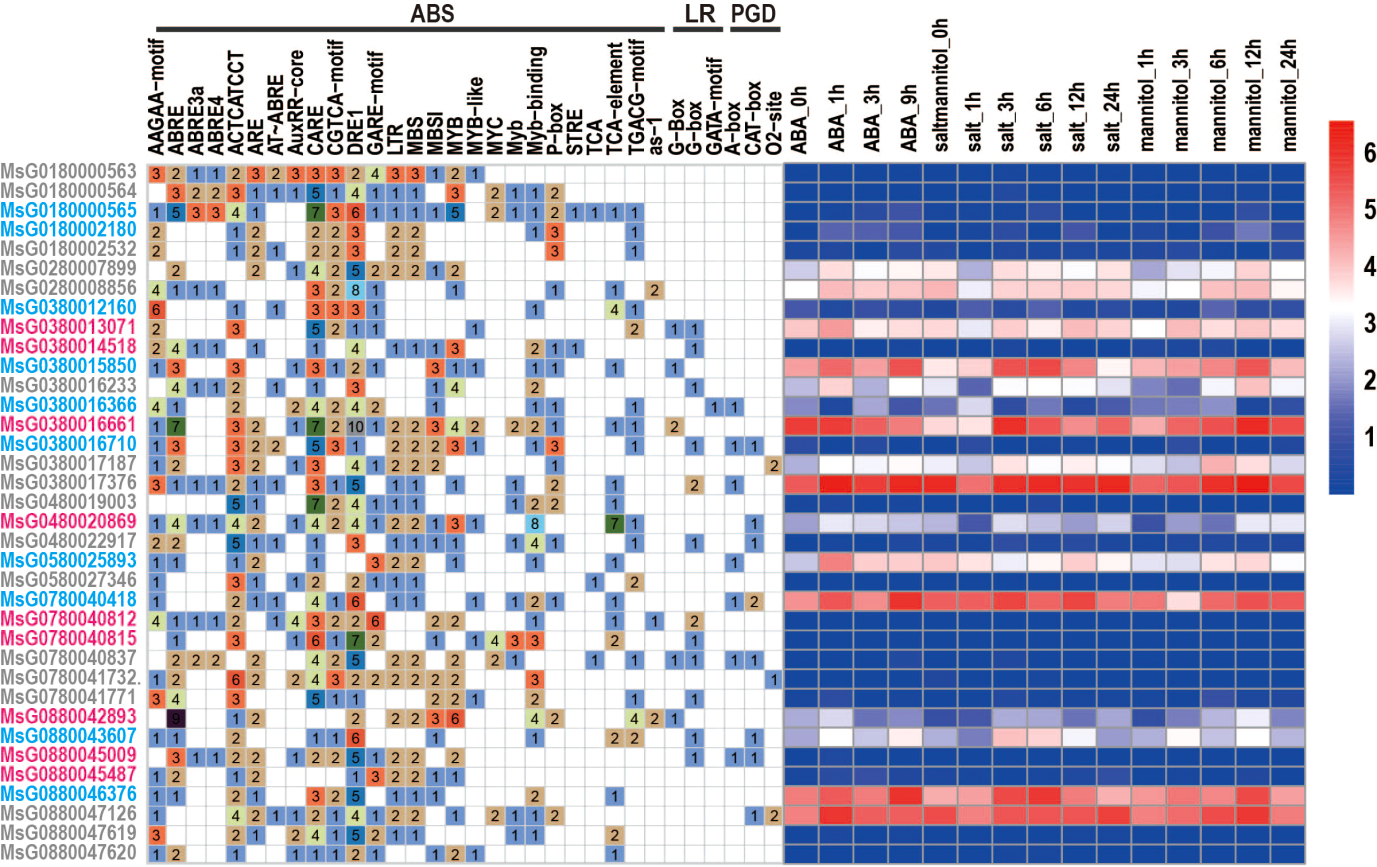
Cis-acting elements in the promoter region of *RRs* and the expression profiles of *RRs* under different stress treatments in alfalfa. ABS: abiotic stress related response elements; LR: light response elements; PGD: plant growth and development response elements. The expression levels of gene are showed in different colors; red and blue represent high and low expression level, respectively. Figure 3. Gene duplications and collinearity analysis.

Cis-acting elements located in the promoter regions are recognized by transcription factors that regulate the spatial and temporal expression patterns of their target genes. To identify these cis-regulating elements in the promoter sequence (2000 bp) of each *RR* gene, we used the PlantCARE online tool and detected many cis-elements in the promoter regions of *RRs* (**Figure 3**). These cis-elements included abiotic stress-related, light, and plant growth and development response elements. As shown in **Figure 3**, almost all *RRs* had abiotic stress-related response elements, such as the ABA-responsive element (ABRE), MeJA-responsive element (TGACG-motif), and salicylic acid-responsive element (TCA-element), suggesting that all *RRs* have the ability to perform their functions under stress. However, not all *RRs* showed high levels of expression under stress conditions. Some genes, including *MsG0180002180*, *MsG0480022917*, *MsG0380016710*, *MsG0180000565*, *MsG0880045009*, *MsG0880045487*, *MsG0780041771*, *MsG0780041732*, *MsG0780040837*, *MsG0780040812*, *MsG0780040815*, *MsG0580027346*, *MsG0180002532*, *MsG0380012160*, *MsG0880047619*, *MsG0480019003*, *MsG0380014518*, *MsG0180000563*, *MsG0180000564*, and *MsG0880047620*, exhibit low expression or no expression under ABA, mannitol, and NaCl treatments (**Figure 3**). This may be caused by heterochromatin-mediated gene silence because most of *RRs* showed telomere-near-distribution. In addition, only 16 and 13 *RRs* had light response elements in plant growth and development in their promoter regions (**Figure 3**), respectively, indicating that some *RRs* are also associated with light response and plant growth and development.

### Gene duplication and collinearity analysis of *RR*s

**Figure 4:**
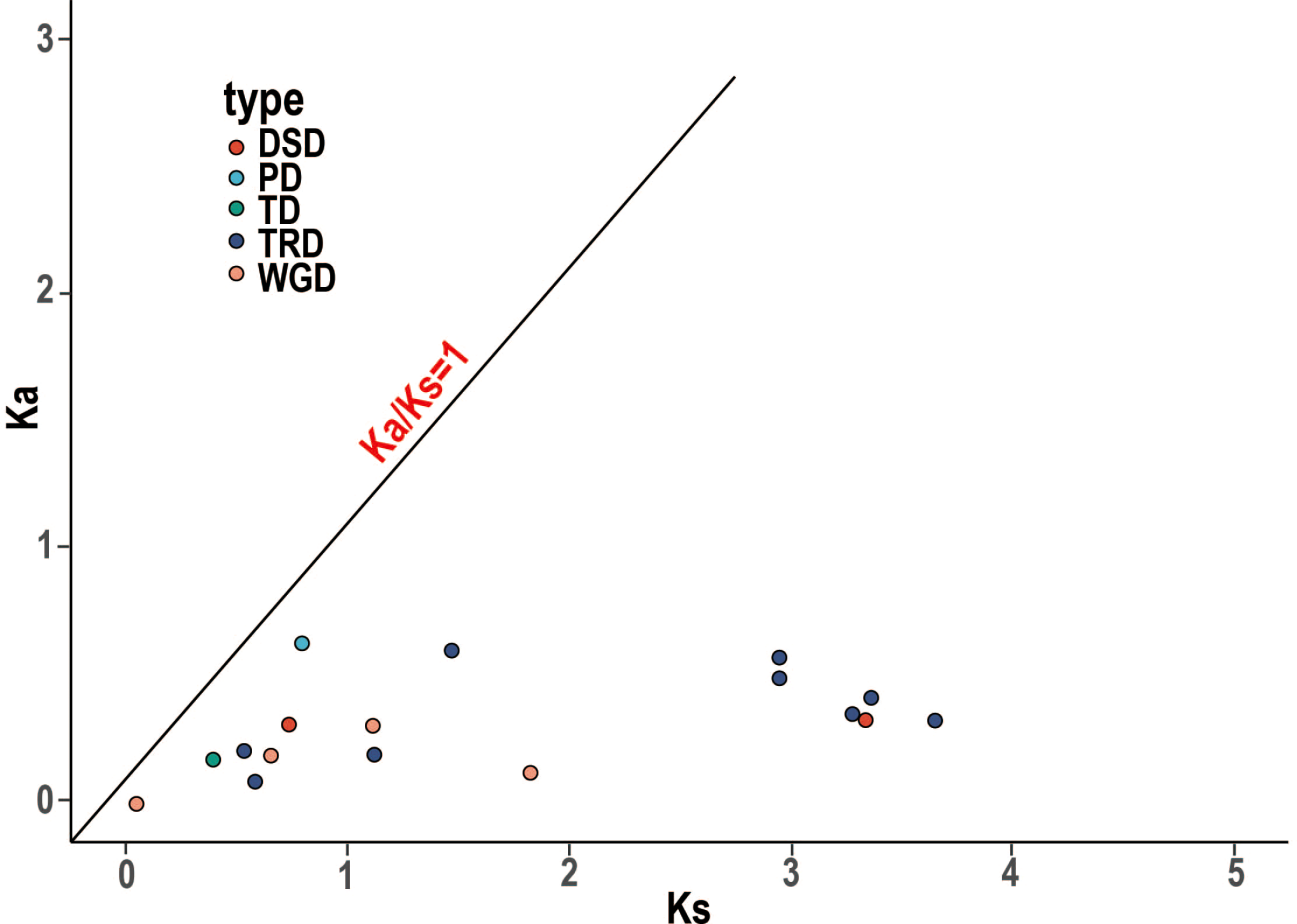
Ka/Ks ratios of duplicated *RR* gene pairs. Gene pairs from different duplication types are indicated by different scatter. The y and x axes denote the Ka and Ks values for each pair and the black line shows a Ka/Ks ratio = 1. The detail of Ka, Ks, and Ka/Ks listed in **Table S2**.

**Figure 5:**
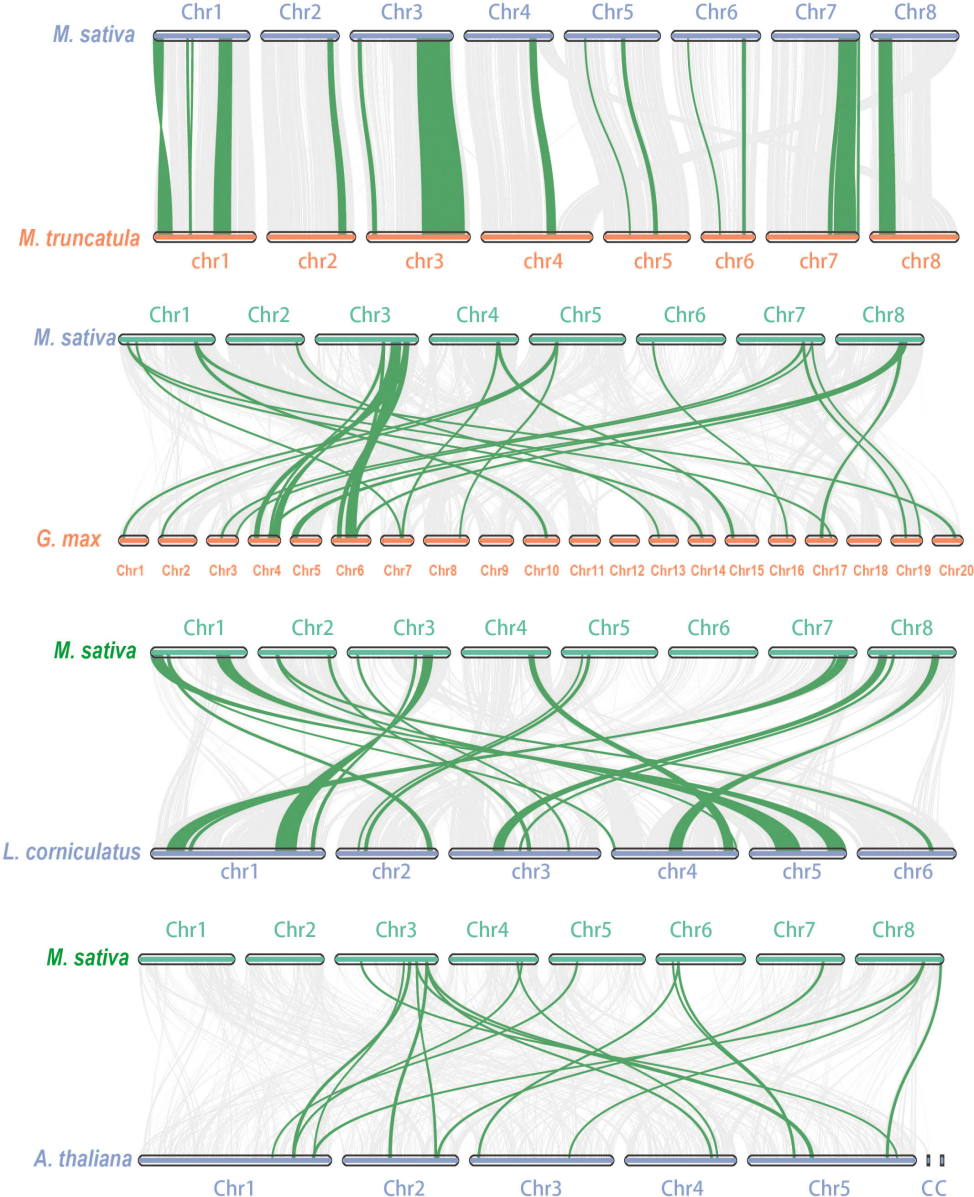
Collinearity analysis between alfalfa and *A. thaliana*/*M. truncatula*/*G. max*/*L. corniculatus*. The gray lines in the background indicate the collinear blocks between alfalfa and *A. thaliana*/*M. truncatula*/*G. max*/*L. corniculatus*, while the blue lines highlight the syntenic *RR* gene pairs.

WGDs along with single gene duplications, such as DSDs, TRDs, PDs, and TDs, are the main driving forces for gene family evolution. In this study, we identified nine TRD events, four WGD events, two DSD events, one TD event, and one PD event (**Figure 4**; **Table 2**) in the *RR* subfamily. To further understand the evolutionary constraints acting on duplicated *RRs*, we calculated Ka (non-synonymous substitution rate), Ks (synonymous substitution rate), and the Ka/Ks ratio for each duplicated *RR* gene pair. Our results showed that all Ka/Ks ratios for the 17 segmentally duplicated gene pairs were < 1 (**Table 2**), indicating that the *RRs* primarily evolved under the influence of purifying selection. To further determine the origin and evolution of *RR* gene family members, four comparative synteny maps were constructed between autotetraploid alfalfa and *A. thaliana*, *Medicago truncatula*, *G. max*, and *Lotus corniculatus*. These results showed that 30 *RRs* of Zhongmu No. 1 were collinear with *G. max*, followed by *M. truncatula* (19), *L. corniculatus* (19), and *A. thaliana* (18). Among these orthologous pairs, seven *RRs* (*MsG0780040418*, *MsG0580025893*, *MsG0880046376*, *MsG0380016710*, *MsG0380016661*, *MsG0380015850*, and *MsG0180000563*) had their corresponding orthologs both in *G. max*, *L. corniculatus*, *M. truncatula*, and *A. thaliana* (**Figure 5**; **Table 3**), suggesting that these genes may play an important role in the evolution of *RRs*. The collinear relationships between the *RRs* of Chr3 and those of the other four plants were significantly greater than those of the other chromosomes.

**Table 2:** The Ka/Ks ratios for duplicated *RRs* paralogous pairs.

Paralogous pairs	Ka	Ks	Ka/Ks	Selective type	Duplicate type
*MsG0780040812- MsG0780040815*	0.6292	0.7942	0.7922	Purifying	PD
*MsG0180000563- MsG0380017376*	0.3061	1.1144	0.2747	Purifying	WGD
*MsG0180000564- MsG0180000565*	0.1734	0.3929	0.4414	Purifying	TD
*MsG0180002180- MsG0180002532*	4.62e-05	0.0462	0.001	Purifying	WGD
*MsG0280007899- MsG0880047126*	0.1929	1.1210	0.1721	Purifying	TRD
*MsG0280008856- MsG0380017376*	0.4923	2.9517	0.1668	Purifying	TRD
*MsG0380015850- MsG0880046376*	0.1217	1.8265	0.0666	Purifying	WGD
*MsG0380016233- MsG0480022917*	0.1892	0.6540	0.2893	Purifying	WGD
*MsG0380016661- MsG0880042893*	0.5735	2.9514	0.1943	Purifying	TRD
*MsG0380016710- MsG0380015850*	0.3520	3.2814	0.1073	Purifying	TRD
*MsG0380017187- MsG0380017376*	0.6010	1.4707	0.4087	Purifying	TRD
*MsG0580025893- MsG0180002180*	0.0871	0.5824	0.1496	Purifying	TRD
*MsG0780040418- MsG0880046376*	0.4157	3.3662	0.1235	Purifying	TRD
*MsG0780041372- MsG0180000563*	0.1807	0.5805	0.3113	Purifying	TRD
*MsG0780041771- MsG0180000563*	0.2075	0.5327	0.3895	Purifying	TRD
*MsG0880043607- MsG0880046376*	0.3266	3.6549	0.0894	Purifying	TRD
*MsG0880045009- MsG0880045487*	0.3109	0.7354	0.4228	Purifying	DSD
*MsG0380012160- MsG0380016366*	0.3283	3.3412	0.0982	Purifying	DSD

**Table 3:** The common syntenic gene pairs of *RRs* among *A. thaliana* and the five legume species.

*M. sativa*	*A. thaliana*	*G. max*	*L. corniculatus*	*M. truncatula*	Subfamily
*MsG0880046376*	*AT1G74890.1*	*Glyma.17G093900.1*	*Lj4g0019308.2*	*Medtr4g106590.1*	A
*MsG0780040418*	*AT3G57040.1*	*Glyma.03G130000.1*	*Lj1g0019360.1*	*Medtr7g490310.2*	A
*MsG0580025893*	*AT1G59940.1*	*Glyma.08G292400.1*	*Lj2g0021492.1*	*Medtr5g036480.1*	A
*MsG0380016710*	*AT2G40670.2*	*Glyma.04G223000.1*	*Lj1g0007309.1*	*Medtr3g093860.1*	A
*MsG0380016661*	*AT5G24470.1*	*Glyma.04G228300.1*	*Lj1g0026375.1*	*Medtr3g092780.1*	P
*MsG0380015850*	*AT3G48100.1*	*Glyma.04G177900.1*	*Lj1g0015853.1*	*Medtr3g078613.2*	A
*MsG0180000563*	*AT2G25180.1*	*Glyma.17G217100.1*	*Lj5g0016527.1*	*Medtr1g013180.1*	B

### Tissue expression patterns of *RRs* in alfalfa

**Figure 6:**
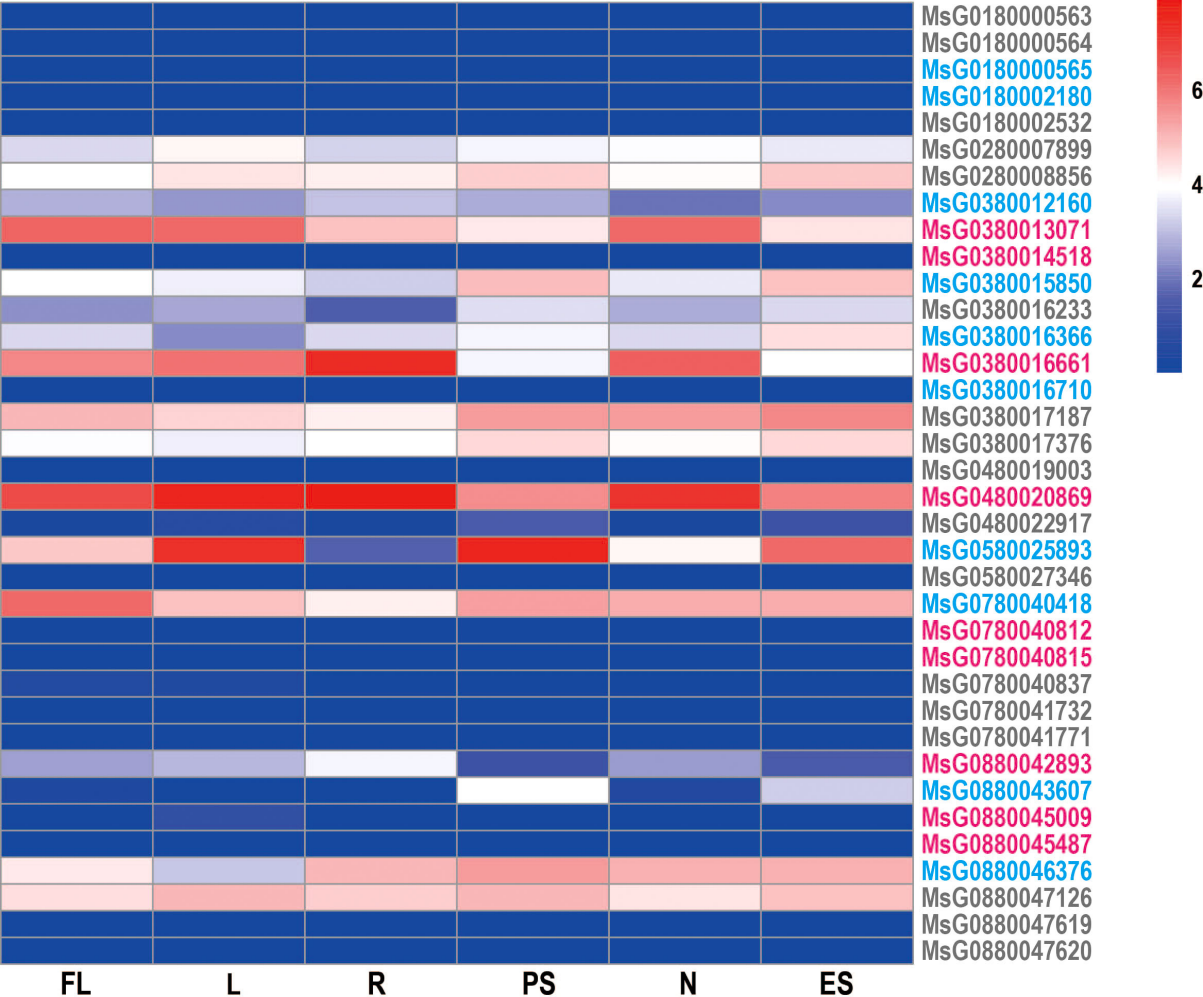
Heatmap displaying the *RRs* expression in various tissues of alfalfa. Transcriptome data from 6 tissues (FL, flower; L, leaf; R, root; PS, pre-elongated stem; N, nodule; ES, elongated stem) were used to construct the expression patterns of *RRs* in alfalfa. The bar to the right of the heat map represents the relative expression values.

Tissue expression profiles are useful in determining the potential roles of target genes and their specific properties in a particular tissue. To gain a deeper understanding of the potential function of *RRs*, we analyzed their expression profiles in six tissues (leaves, flowers, pre-elongated stems, elongated stems, roots, and nodules) using RNA-sequencing data from alfalfa and found that the expression patterns of *RRs* were distinct. Most *RRs* showed a constitutive expression pattern, but nearly half of the genes were expressed at low levels in all tissues. This may be caused by the telomere-near-distribution due to the heterochromatin-mediated epigenetic regulation of gene expression (Matzke et al., 2009; To et al., 2011). Furthermore, *MsG0380013071*, *MsG0280008856*, *MsG0880047126*, *MsG0480020869*, and *MsG0380017187* were highly expressed in all six tissues. Interestingly, we also observed tissue-specific expression patterns of some *RRs*. For example, *MsG0880043607* was expressed only in the stem, while *MsG0580025893* was highly expressed in leaves, stems, and flowers (**Figure 6**; **Figure S2**).

## Discussion

In response to abiotic stress, RRPs obtain phosphoric groups from histidine phosphate transfer proteins to stimulate the transcription of downstream functional genes. Previous studies have reported that *RRs* in various plants, such as *A. thaliana*, *O. sativa*, *Pyrus bretschneideri*, *Prunus persica*, *Fragaria vesca*, and *Brassica rapa* ssp. *Pekinensis* (Liu et al., 2014; Bhaskar et al., 2021), are crucial for the plant response to abiotic stress (Imamura et al., 1999), which can be applied in alfalfa molecular breeding for drought tolerance. It is also important to elucidate the *RR*-related genes and their functions in alfalfa and other legumes. This will expand our knowledge of *RRs* and provide a foundation for further investigation of their specific roles and functions. In this study, 37 *RRs* were identified in alfalfa, which is approximately equivalent to the *RR* family size in *A. thaliana* (32 genes) and *O. sativa* (36 genes) (Ramírez-Carvajal et al., 2008). The close relationship between *RRs* of *A. thaliana* and alfalfa, as observed in the phylogenetic tree, suggests that the functions of these *RRs* in alfalfa may be similar to those observed in *A. thaliana*. Based on evolutionary relationships and structural specificity, the 37 *RRs* can be clustered into three subfamilies: type-A, type-B, and type-P *RRs* (**Figure 1B**). Protein domain analysis revealed that the conserved motifs within each subfamily were similar, but those among different subfamilies were significantly different. Specifically, motifs 1 and 5 were present in the members of type-P RRPs, while motifs 1, 2, and 5 were present in the members of the type-A RRPs, and all motifs were present in most members of type-B RRPs (**Figure 1B**). These differences in characteristics among the different subfamilies may indicate the diverse functions of the *RR* family members in alfalfa.

Gene duplication is a fundamental source of new genes in the evolutionary process, facilitating the successful evolution of genes and contributing to the rapid expansion of gene families (Van de Peer et al., 2009; Wang et al., 2017). *RR*-related duplication events have been observed in many species, including *A. thaliana*, *Malus domestica*, and *Gossypium* species (Li et al., 2017). Our gene duplication analysis revealed that *RRs* in alfalfa also experienced different types and numbers of gene duplication events. In *Gossypium* species, no tandem duplication gene pairs were detected in the *RR* gene family, and WGD seemed to contribute more to alfalfa *RR* gene family expansion than single gene duplication events; however, our analysis documented the presence of TD events and revealed that TRD events, not WGD events, played a major role in the expansion of the *RR* gene family in alfalfa (**Figure 4**). These conflicting findings may be attributed to the independent evolution of genes.

*RRs* have been reported to be involved in stress resistance and response in various plants, such as *A. thaliana* (Nakamichi et al., 2016) and *O. sativa* (Karan et al., 2009; Rehman et al., 2022), and several *RRs* in alfalfa were also found to respond to different types of abiotic stresses (**Figure 3**). For example, *MsG0380016233*, *MsG0580025893*, *MsG0780040418*, *MsG0280008856*, *MsG0380013071*, *MsG0380017187*, *MsG0480020869*, and *MsG0280007899* were significantly up-regulated by ABA treatment, some of which were confirmed by qRT-PCR analysis (**Figure S3**). Cis-element analysis indicated that ABREs were located in the promoter regions of these genes, and subcellular localization predicted analysis revealed that they were only localized in the nucleus (**Figure 2B**). Taken together, these results suggest that these genes may be directly regulated by ABA in response to drought stress (**Figure 3**) and should be transferred into the nucleus to regulate the expression of some genes in response to ABA stimuli. Interestingly, MsG0580025893 was predicted to be distributed in both the nucleus and chloroplast, and was highly expressed in leaves, stems, and flowers (**Figure 2B**; **Figure 6**), suggesting that it may function in abiotic stress by regulating both nuclear and chloroplast gene expression to enhance photosynthesis. In addition, we also identified several *RRs*, including *MsG0180002180*, *MsG0480022917*, *MsG0380016710*, *MsG0180000565*, *MsG0880045009*, *MsG0880045487*, *MsG0780041771*, *MsG0780041732*, *MsG0780040837*, *MsG0780040812*, *MsG0780040815*, *MsG0580027346, MsG0180002532*, *MsG0380012160*, *MsG0880047619*, *MsG0480019003*, *MsG0380014518*, *MsG0180000563*, *MsG0180000564*, and *MsG0880047620*, which were not expressed under ABA, NaCl, and mannitol treatments (**Figure 3**). Interestingly, ABREs were still detected in the promoter region of *MsG0180000565*. This could be partly explained by the presence of other regulatory mechanisms, such as heterochromatin-mediated gene silence, that compete with ABA induction during transcription (Peng et al., 2008). *RRs* also play important roles in plant immune response and disease resistance (Cheval et al., 2017; Bacete et al., 2020). In line with this, we found that some genes, such as *MsG0780040815* and *MsG0380016661*, have gibberellin, salicylic acid, and methyl jasmonate response elements in their promoter regions (**Figure 3**).

Circadian rhythms are produced by the internal clock or oscillator of many organisms and control the timing of genetic, metabolic, and physiological processes, including photosynthesis (Dodd et al., 2005; Chow and Kay, 2013), growth rate, and flowering time (Woelfle et al., 2004). Photoperiodic regulation of the flowering period is one of the most common circadian events (Yanovsky and Kay, 2003). In *A. thaliana*, the *APRR* family has been reported to be associated with the circadian clock (Murakami et al., 2004). The expression patterns of some orthologs of *AtPRRs* in *Populus* exhibit circadian waves (Liu et al., 2013), but the circadian rhythm function of alfalfa type-P *RRs* has not yet been reported. Notably, photoperiod-responsive elements, including G-box, GATA-motif, and methyl jasmonate, were also detected in the promoter region of type-P *RRs*, suggesting that members of the *RR* family may also be related to the circadian rhythm (**Figure 3**). Subcellular localization analysis of *A. thaliana* and *Glycyrrhiza uralensis* revealed that type-P RRPs are mainly localized in the nucleus (Fujiwara et al., 2008). However, in our predicted results, type-P RRPs were found to exist not only in the nucleus but also in the cytoplasm, mitochondria, and chloroplasts (**Figure 2B**). This suggests that type-P *RRs* in alfalfa have roles other than the maintenance of circadian rhythm, which require further exploration future studies.

## Conclusion

In this study, we comprehensively and systematically analyzed the response regulator gene family in the autotetraploid-cultivated alfalfa genome. We identified 37 *RR* family genes unevenly distributed across eight chromosomes in the alfalfa genome and further grouped them into three subfamilies, type-A, type-B, and type-P *RRs*, based on their evolutionary relationship, structural specificity, and degree of conservation. We also analyzed the physicochemical properties, phylogenetic relationships, exon–intron structures, conserved motifs, chromosomal location, gene duplication, cis-regulatory elements, and tissue-specific expression patterns of these genes. Our findings shed light on the significant roles of alfalfa *RR* gene family in hormone induction and abiotic stress tolerance and have great scientific and practical application in alfalfa molecular breeding.

## Data availability statement

The original contributions presented in the study are included in the article/**Supplementary Material**. Further inquiries can be directed to the corresponding authors.

## Author contributions

YQ, XH and LF designed the experiments and wrote the manuscript. YQ, SL, JZ, MT, XW, TL analyzed the data. LF, ZL and ZPL edited the manuscript. All authors contributed to the article and approved the submitted version.
