# Supplementary material for: Genome-wide identification and expression analysis of the response regulator gene family in alfalfa (Medicago sativa L.) reveals their multifarious roles in stress response
Source: Front Plant Sci. 2023 Mar 14;14:1149880. doi: 10.3389/fpls.2023.1149880 (PMC10043395; doi:10.3389/fpls.2023.1149880)
Supplement: Supplementary file 1 [file DataSheet_1.docx]

**Genome-wide identification and expression analysis of the response regulator** **gene family in** **alfalfa (*Medicago sativa*** **L.) reveals their multifarious roles in stress response**

Yuqing Qiang^1†^, Xiaojuan He^1†^, Zhen Li^2^, Siqi Li^1^, Jia Zhang^1^, Tao Liu^1^, Mamateliy Tursunniyaz^1^, Xinyu Wang^1^, Zhipeng Liu^1,^* and Longfa Fang^1,^*

^1^State Key Laboratory of Herbage Improvement and Grassland Agro-ecosystems, College of Pastoral Agriculture Science and Technology, Lanzhou University, Lanzhou 730020, China

^2^National Engineering Laboratory for VOCs Pollution Control Material & Technology, University of Chinese Academy of Sciences, Beijing, 100049, China

^†^These authors contributed equally to this work and share first authorship.


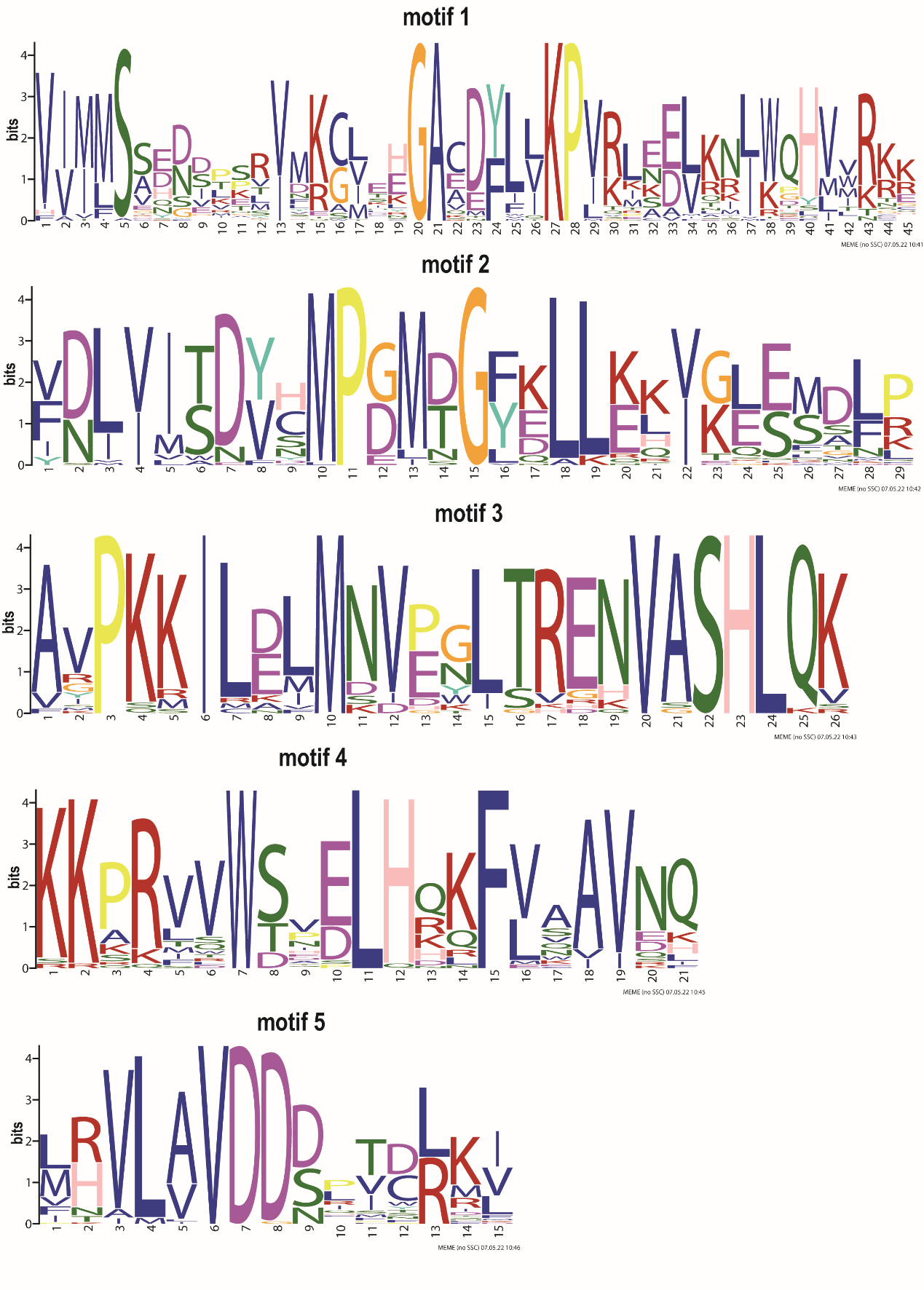


**Figure S1. The motif structures of the *RRs* in alfalfa**

**
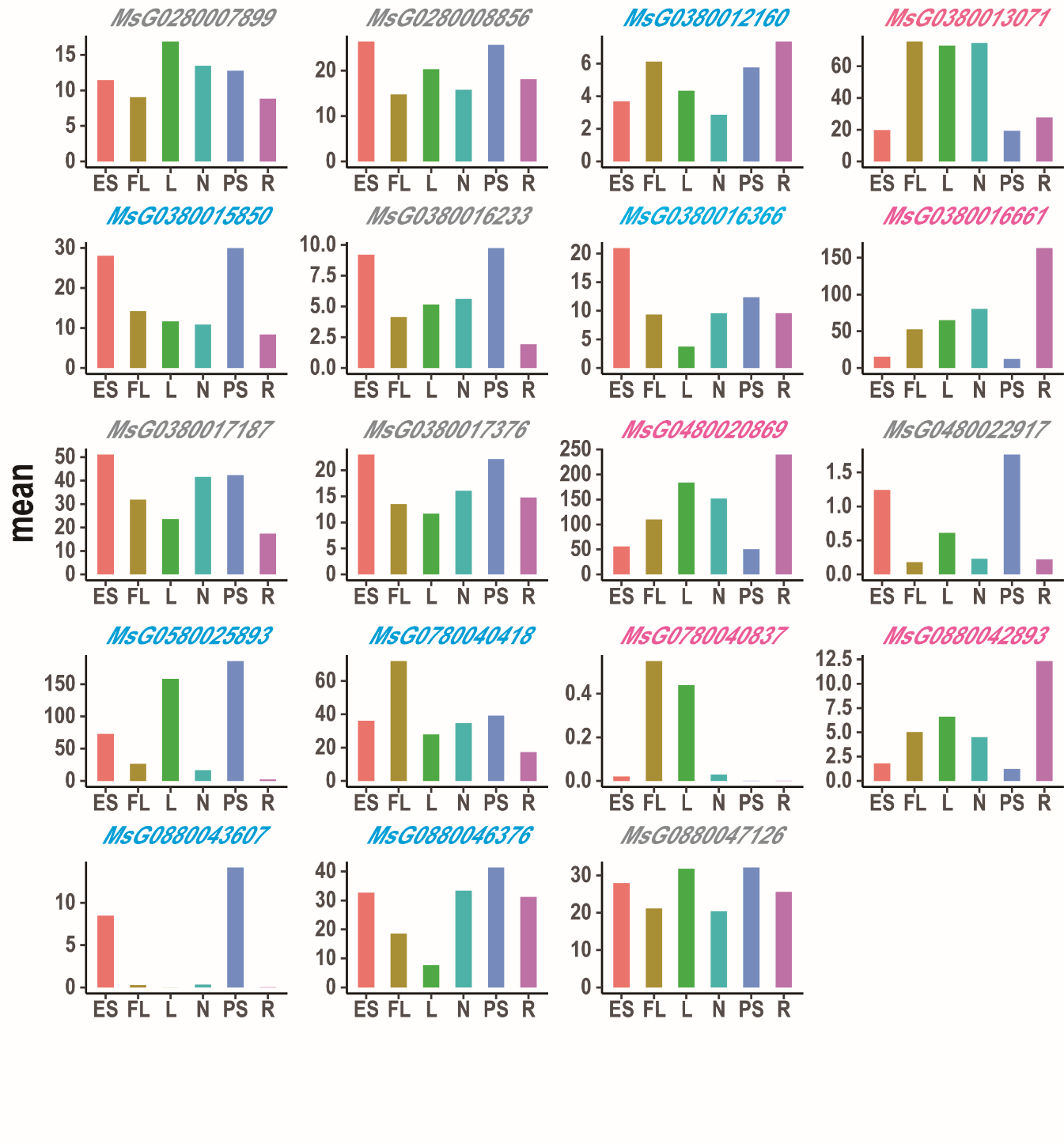
**

**Figure S2. Relative expression of *RRs* in different tissues of alfalfa.** ES: elongated stem; FL: flower; L: leaf; N: nodule; PS: pre-elongated stem; R: root.


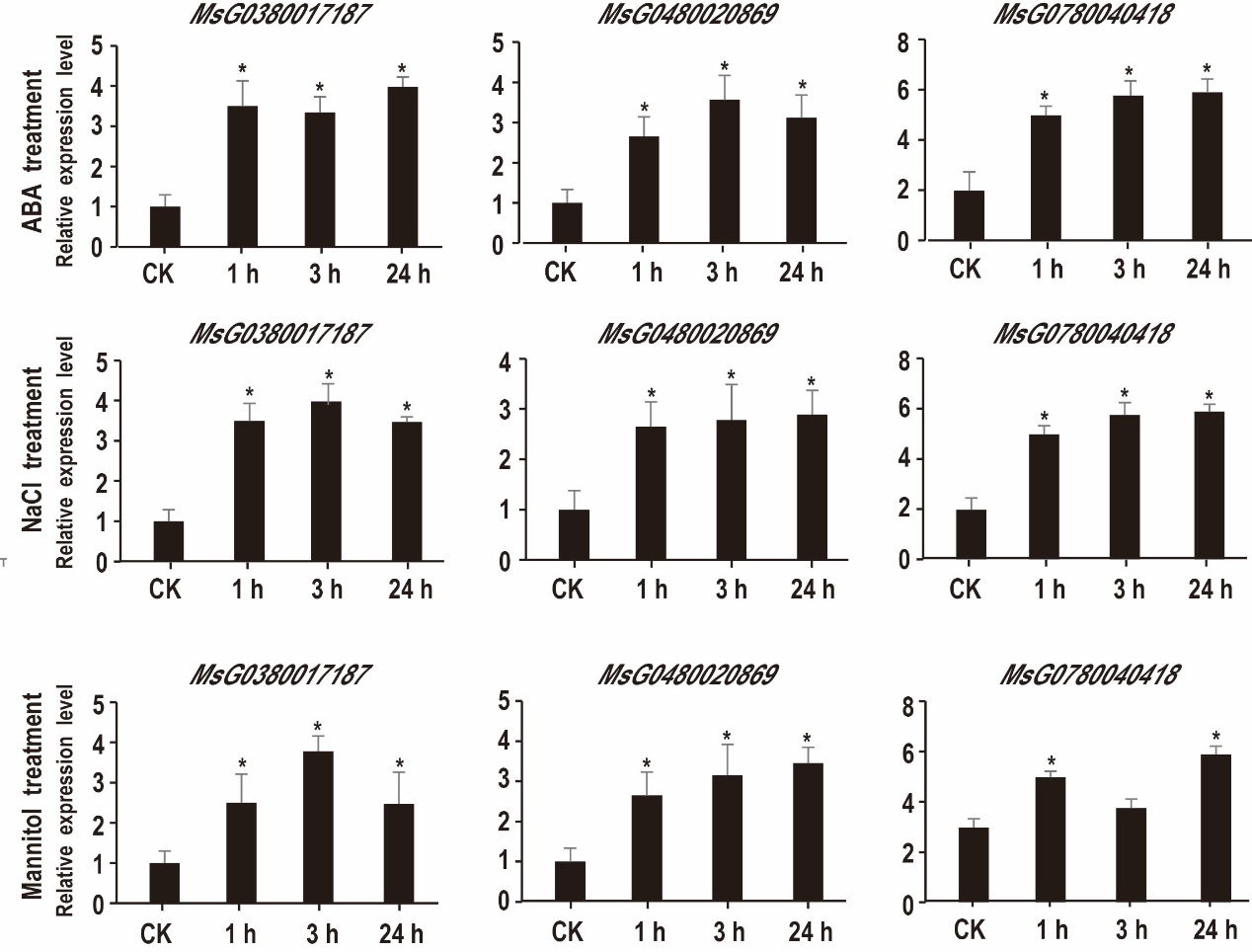


**Figure S3. qRT-PCR analysis of some *RRs* under ABA, mannitol, and NaCl treatments.**

**Table S1 Sequences of forward and reverse primer pairs for real-time quantification PCR**

| **Gene** | **Primers sequences** |
| --- | --- |
| *MsG0380017187*-F | CTCCTTCTCTCTTGCTTTTGGAAA |
| *MsG0380017187*-R | GACAGGAAGATCCATTTCAAGTCCT |
| *MsG0780040418*-F | ATGGGAATGGCTGCAGAGTCA |
| *MsG0780040418*-R | GCCTTGCTACCAGAATCAACTGTAG |
| *MsG0480020869*-F | ATAATGTTGGGAAACAAAGTTTGGC |
| *MsG0480020869*-R | ATTTCCAGTTGATGATTCAGACCC |
| *Actin*-F | ACTGGAATGGTGAAGGCTGG |
| *Actin*-R | TGACAATACCGTGCTCAATGG |
